# 3D-printed sound absorbing metafluid inspired by cereal straws

**DOI:** 10.1038/s41598-019-44891-z

**Published:** 2019-06-11

**Authors:** W. Huang, L. Schwan, V. Romero-García, J.-M. Génevaux, J.-P. Groby

**Affiliations:** Laboratoire d’Acoustique de l’Université du Mans, LAUM - UMR CNRS 6613, Le Mans Université, Avenue Olivier Messiaen, 72085 Le Mans Cedex 9, France

**Keywords:** Bioinspired materials, Acoustics, Characterization and analytical techniques

## Abstract

Used as building biomaterials for centuries, cereal straws are known for their remarkable acoustic performances in sound absorption. Yet, their use as fibrous media disregards their internal structure made of nodes partitioning stems. Here, we show that such nodes can impart negative acoustic bulk modulus to straw balls when straws are cut on either side of a node. Such metafluid inspired by cereal straws combines visco-thermal diffusion with strong wave dispersion arising from quarter-wavelength resonances within straws. Large spectral bandgaps and slow sound regimes are theoretically predicted and experimental data from impedance tube measurements on an idealised 3D-printed sample layer are in good agreement with the theoretical model. Perfect absorption is achieved at wavelengths 13 times larger than the thickness of the metafluid layer, and slow sound entails an increased density of states causing a cascade of high absorption peaks. Such features could lead cereal straws to serve as cheap acoustic bio-metamaterials.

## Introduction

Recent developments in manufacturing techniques, such as Fused Filament Fabrication and Stereolithography technologies, have considerably broadened the range of possible architectures for artificial materials^1^. Advanced material properties have subsequently been tailored in many fields of physics, among which pentamode mechanics^2^, acoustic rainbow trapping^3^, metasurface retroreflector^4^, or microwave atomic clock^5^. From this perspective, biological systems can be a valuable source of inspiration: idealised synthetic replicas mimicking fauna and flora^6^, spider-web structures^7^, or sperm-cell motion^8^ have demonstrated astonishing performances in photonics, lattice mechanics, and micro-robotics.

**Figure 1:**
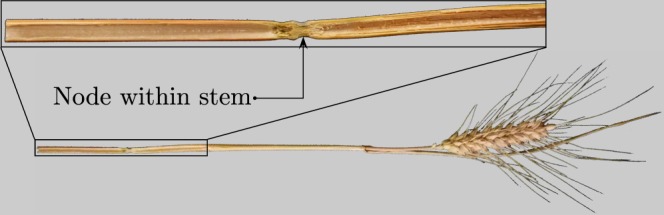
Photograph of a wheat straw and details of the inner node partitioning the stem into separated tubular segments.

Particularly interesting in acoustic and thermal insulation are cereal straws such as wheat, reed or rattan. They have been used as building materials for centuries due to their large availability throughout the world. Previous empirical^9^, phenomenological^10^, and theoretical^11^ studies showed that viscous and thermal diffusion through the arrangement of stems play a dominant role in acoustic performances. Attention was mainly paid to the effects of the length, concentration, and relative orientations of the straws on sound absorption. While arrays of straws were usually idealised as classical visco-thermal fluids made of either solid or completely hollow straws, closer examination of cereal straws in Fig. 1 reveals that nodes within the stem partition the straw into tubular segments. Therefore, when cutting straws on either side of a node, the resulting pieces are neither solid obstacles in the path of the acoustic wave, nor open hollow straws channelling the flow, but rather double quarter-wavelength resonators (QWR) separated by the node.

**Figure 2:**
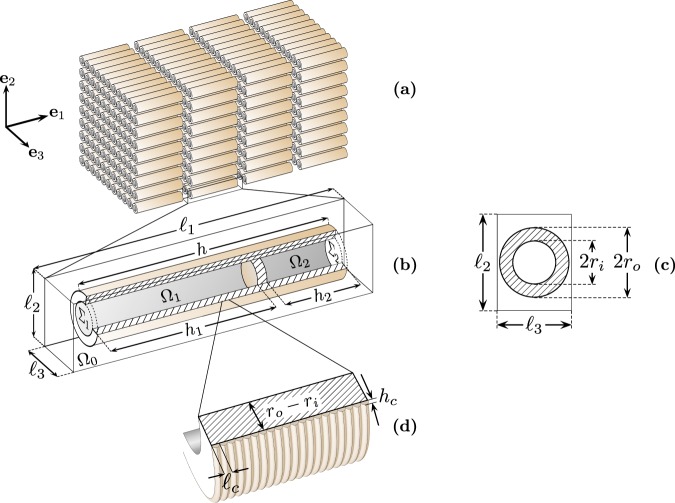
Schematic of the idealised metafluid inspired by cereal straws. (**a**) Representation of the ${\rm{\Omega }}$-periodic arrangement of the double Quarter-Wavelength Resonators (QWRs) elements. (**b**) Details of the unit cell. (**c**) Details of the QWR opening. (**d**) Zoom at the corrugation of the walls.

Based on this observation, we designed a metafluid inspired by the cereal straws (MCS) made of the periodic repetition of double QWR, see Fig. 2. We theoretically show that their collective resonances entail an effective negative bulk modulus in the MCS around the QWR fundamental frequency resulting in both band gap opening and strong wave dispersion. In particular, the strong dispersion induced by the double QWR below its fundamental resonance generates a slow propagation of sound over a wide band in the low frequencies. Therefore, the Fabry-Perot resonances of a MCS layer appear in the deep subwavelength regime and its associated density of states is increased. Combining these effects with the viscous and thermal diffusion tailored by the concentration of double QWR, each low quality factor mode of the MCS layer can yield perfect absorption peaks due to a critical coupling between the inherent losses and the energy leakage of the MCS layer^12^. The theoretical predictions are supported experimentally by data from impedance tube measurements on prototypes fabricated by Fused Filament Fabrication. This manufacturing technique induces corrugation at the walls of the straws which has been advantageously used to enhance further attenuation in the manufactured sample. Perfect absorption of sound for wavelengths in air 13 times larger than the layer thickness has been achieved with large absorption value below the first resonance of the double QWR.

## Idealisation of the Cereal Straw Arrangement

The cereal straw arrangement is idealised by a three-dimensional ${\rm{\Omega }}$-periodic repetition of straight double QWR, as shown in Fig. 2. The unit cell ${\rm{\Omega }}$ is a rectangular cube with the dimensions ${\ell }_{1}\times {\ell }_{2}\times {\ell }_{3}$ in the directions of the Cartesian coordinate system $({{\bf{e}}}_{1},{{\bf{e}}}_{2},{{\bf{e}}}_{3})$ containing a double QWR. The axis of each double QWR is oriented along the direction **e**_1_ and consecutive stacks of straws are separated by an air-gap to permit air to flow inside the straws. Each double QWR has the outer radius *r*_*o*_, the inner radius ${r}_{i} < {r}_{o}$, and the finite length $h\le {\ell }_{1}$. The volume of the double QWR is split by a rigid node into two cylindrical ducts ${{\rm{\Omega }}}_{1}$ and ${{\rm{\Omega }}}_{2}$ with the lengths *h*_1_ and *h*_2_ and the outside apertures ${{\rm{\Sigma }}}_{1}$ and ${{\rm{\Sigma }}}_{2}$ respectively. Denoting by ${{\rm{\Omega }}}_{0}$ the air domain of the unit cell outside the straw, the filling fractions ${\varphi }_{0}=|{{\rm{\Omega }}}_{0}|/|{\rm{\Omega }}|$, ${\varphi }_{1}=|{{\rm{\Omega }}}_{1}|/|{\rm{\Omega }}|$ and ${\varphi }_{2}=|{{\rm{\Omega }}}_{2}|/|{\rm{\Omega }}|$ are defined and the total porosity of the arrangement is $\varphi ={\varphi }_{0}+{\varphi }_{1}+{\varphi }_{2}$. Besides, the outside walls of the straw are supposed to be corrugated periodically with the period ${\ell }_{c}$ in the direction **e**_1_ and the semi-elliptic profile of height *h*_*c*_ in a longitudinal cross-section, see Fig. 2(d). Although, this corrugation is not fundamental to the singular behaviour of the metafluid, it is an inherent side-effect of the layer-by-layer 3D-printing process using Fused Filament Fabrication technology. It must be accounted for theoretically in the present case of tightly-packed straw arrangements, for which the corrugation height *h*_*c*_ is of the same order as the spacing ${\ell }_{2}-2{r}_{o}$ between two neighbouring straws.

## Sound Propagation in the MCS

The propagation of air-borne acoustic waves in the MCS is studied under ambient conditions in the linear harmonic regime at frequencies $\omega$ with the implicit time factor ${e}^{-{\rm{i}}\omega t}$. Under the assumption that lattice sizes ${\ell }_{1}$, ${\ell }_{2}$, ${\ell }_{3}$ are sufficiently small compared to the wavelength ${\lambda }_{e}=2\pi$/${k}_{e}$ in air (typically below the Bragg limit ${\ell }_{1}\le {\lambda }_{e}$/2), the idealised system is expected to behave as a metafluid, that is an effective homogeneous medium with strong wave dispersion induced by microstructural QWR resonances. The effective pressure *P* and particle velocity **V** in the MCS are governed by the following equations of mass conservation and generalized Darcy law, see Section Methods for details,

1 $$\nabla \cdot {\bf{V}}={\rm{i}}\omega P/B,\,{\rm{and}}\,{\bf{V}}=-\,{\bf{K}}\nabla P/\eta ,$$

where *η* is the viscosity in air and where both the effective bulk modulus *B* and the Darcy permeability tensor **K** are complex-valued and frequency dependent. In particular, the Darcy tensor is diagonal in the coordinate system $({{\bf{e}}}_{1},{{\bf{e}}}_{2},{{\bf{e}}}_{3})$ due to the symmetries of the unit cell ${\rm{\Omega }}$,

2 $${\bf{K}}={K}_{1}\,{{\bf{e}}}_{1}\otimes {{\bf{e}}}_{1}+{K}_{2}\,{{\bf{e}}}_{2}\otimes {{\bf{e}}}_{2}+{K}_{3}\,{{\bf{e}}}_{3}\otimes {{\bf{e}}}_{3},$$

where the principal permeabilities *K*_*n*_ with $n\in \{1,2,3\}$ are expressed by the Johnson formula^13^, and the homogenisation theory^14,15^, see Section Methods. The effective bulk modulus *B* satisfies the following association rule when normalised by the bulk modulus *B*_*e*_ of air,

3 $${B}^{-1}={B}_{e}^{-1}({\varphi }_{0}{\beta }_{0}+{\varphi }_{1}{\beta }_{1}+{\varphi }_{2}{\beta }_{2}),$$

where *β*_0_ is the compressibility factor considering the thermo-acoustic phenomena in the domain ${{\rm{\Omega }}}_{0}$, and *β*_1_ and *β*_2_ are apparent compressibility factors induced by the resonances in the domains ${{\rm{\Omega }}}_{1}$ and ${{\rm{\Omega }}}_{2}$. While *β*_0_ is given by the Johnson-Lafarge model^13,16^ and the homogenisation theory^14,15^, the apparent compressibility factors *β*_1_ and *β*_2_ account for the fact that the QWRs act as secondary acoustic sources in the medium, see Section Methods. They can be expressed in the form,

4 $${\beta }_{j}={(\frac{{B}_{j}}{{B}_{e}}\frac{{k}_{j}{h}_{j}}{\tan ({k}_{j}{h}_{j})}-\mu {k}_{e}^{2}{r}_{i}{h}_{j})}^{-1},\,j\in \{1,2\},$$

where ${k}_{j}=\omega /\sqrt{{B}_{j}/{\rho }_{j}}$ is the wavenumber in the domain ${{\rm{\Omega }}}_{j}$, with the effective density ${\rho }_{j}$ and bulk modulus *B*_*j*_ in ${{\rm{\Omega }}}_{j}$ being derived from the Zwikker & Kosten model^17^. Moreover, the dimensionless factor $\mu =1.2$ in Eq. (4) corrects the radiation impedance at the aperture ${{\rm{\Sigma }}}_{j}$.

Hence, the quarter-wavelength resonance of the duct ${{\rm{\Omega }}}_{j}$ can cause *β*_*j*_ and thus the bulk modulus *B* to become negative^18,19^, which offers the possibility to open non-propagative band-gaps and generate slow sound regimes. Moreover, since the inner resonances only affect the mass conservation, non-propagative band-gaps are opened for any direction of propagation in the MCS, regardless of the anisotropy of the Darcy tensor.

## Effective Fluid Parameters of the MCS

The MCS with the following geometry is considered,

5 $$\{\begin{array}{lll}{\ell }_{1}=41\,{\rm{mm}}, & {\ell }_{2}=8.4\,{\rm{mm}}, & {\ell }_{3}=8.4\,{\rm{mm}},\\ h=40\,{\rm{mm}}, & {h}_{1}=30\,{\rm{mm}}, & {h}_{2}=10\,{\rm{mm}},\\ {r}_{o}=4.0\,{\rm{mm}}, & {r}_{i}=3.8\,{\rm{mm}}, & \\ {\ell }_{c}=209\,\mu {\rm{m}}, & {h}_{c}=77\,\mu {\rm{m}}. & \end{array}$$

**Figure 3:**
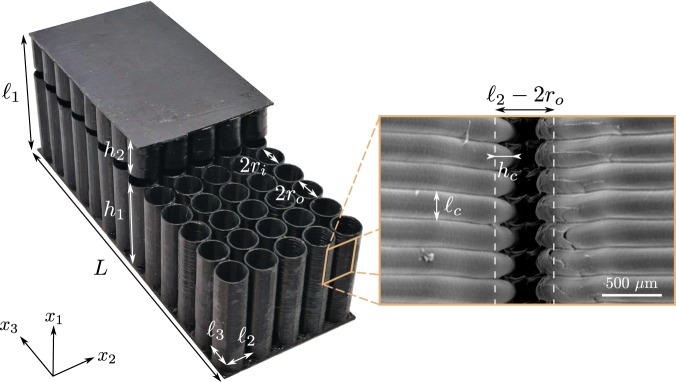
Cut view of the 3D-printed prototype manufactured by means of Fused Filament Fabrication. The inset depicts an Electron Scan Microscopy (ESM) cliché of the walls of two neighbouring 3D printed straws obtained after gold deposition at the surface of the plastic walls.

A photograph of the 3D-printed MCS prototype manufactured by means of Fused Filament Fabrication is shown in Fig. 3(a). To characterise the corrugation at the outer walls of the straws and estimate ${\ell }_{c}$ and *h*_*c*_, a cliché by Electron Scan Microscopy is presented in Fig. 3(b). It bears testament to the fact that the corrugation height *h*_*c*_ is of the same order as the spacing ${\ell }_{2}-2{r}_{o}\approx 400\,\mu {\rm{m}}$ between two neighbouring straws. While the corrugation parameters ${\ell }_{c}$ and *h*_*c*_ are prescribed by the 3D-printer, the other geometrical parameters in Eq. (5) result from an optimisation procedure presented in Section Methods. It is worth noting here that such geometry with ${\ell }_{2}={\ell }_{3}$ entails the orthotropy of the Darcy tensor, with the principal permeabilities *K*_2_ and *K*_3_ being equal in Eq. (2).

**Figure 4:**
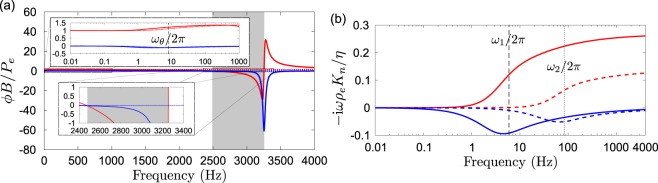
Effective parameters. (**a**) Real (red line) and imaginary (blue line) parts of the normalized bulk modulus for the MCS (solid line) and for the FCS (dashed line). Zoom at negative bulk modulus region (shadowed area) in the lower inset, and at the transition between isothermal and adiabatic regimes around characteristic frequency *ω*_*θ*_/2*π* in the upper inset. (**b**) Real (red lines) and imaginary (blue line) parts of the normalized Darcy permeabilities for the two principal directions *e*_1_ (solid lines) and *e*_2_ (dashed lines), and position of the characteristic frequencies *ω*_1_/2*π* and *ω*_2_/2*π*.

Figure 4(a,b) represent the effective normalised parameters $\varphi B$/${P}_{e}$ and $-{\rm{i}}\omega {\rho }_{e}{K}_{n}$/$\eta$ with $n\in \{1,2\}$ for the MCS, where *P*_*e*_ is the atmospheric pressure and ${\rho }_{e}$ the density of air. For the sake of comparison, both quantities in the absence of the QWR, that is when ${r}_{i}=0$ and hence ${\varphi }_{j}=0$ for $j\in \{1,2\}$, are also plotted. This latter system can be seen as a equivalent anisotropic fluid inspired by cereal straws (FCS)^20^. As shown in Fig. 4(a) and emphasised in the lower inset, the fundamental resonance of the double QWR induces negative bulk modulus *B* over the broad frequency range $[2460,3254]\,{\rm{Hz}}$. The low frequency limit of the bulk modulus is also magnified in the upper inset, which shows the transition between the isothermal and adiabatic regimes of the MCS around the characteristic thermal frequency *ω*_*θ*_/2*π* ≈ 8.24 Hz^13,16^. Compared to the QWR resonance, this thermal transition occurs at much lower frequencies and with much smaller impact on the effective bulk modulus. Figure 4(b) represents the complex and frequency dependent Darcy permeabilities of the MCS. Note that the Darcy permeabilities of the FCS are the same as those of the MCS. Differences between *K*_1_ and *K*_2_ clearly show the anisotropic behaviour of the system. The frequencies *ω*_1_/2*π* ≈ 6 Hz and *ω*_2_/2*π* ≈ 83 Hz characterise the transition between the viscodiffusive regime and the inertio-propagative regime in the principal directions **e**_1_ and **e**_2_^13,16^.

## Dispersion Relation in the MCS

**Figure 5:**
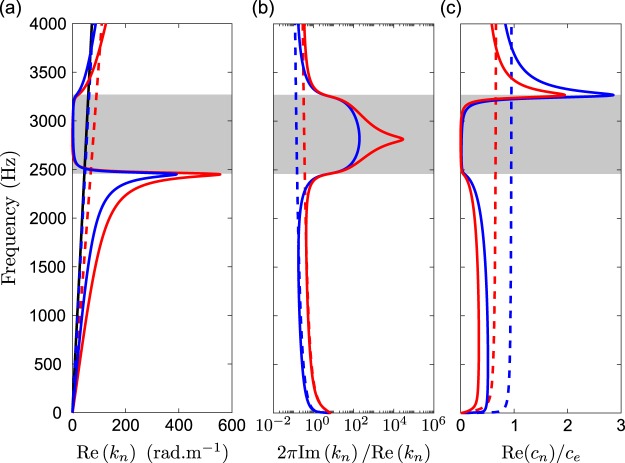
Dispersion relation along the principal directions **e**_1_ (blue lines) and **e**_2_ (red lines): (**a**) real and (**b**) normalized imaginary parts of the wavenumber and (**c**) normalized effective wave speed for the MCS (continuous lines), for the FCS (dashed lines) and for the air (black continuous line).

To show the effects of the effective fluid parameters on wave propagation, the dispersion relation in both the MCS and the FCS is studied. In each principal direction **e**_*n*_ with *n* ∈ {1, 2, 3}, the wavenumber *k*_*n*_ and the sound speed *c*_*n*_ are given by *k*_*n*_ = *ω*/*c*_*n*_ and ${c}_{n}=\sqrt{-{\rm{i}}\omega {K}_{n}B/\eta }$, where *c*_2_ = *c*_3_ due to the orthotropy in the system. The real and imaginary parts of the wavenumbers ${k}_{n}^{MCS}$ in the MCS, ${k}_{n}^{FCS}$ in the FCS and *k*_*e*_ in air are shown in Fig. 5(a,b) while the normalized sound speeds Re(*c*_*n*_)/*c*_*e*_ are shown Fig. 5(c), where *c*_*e*_ is the sound speed in air.

First of all, the anisotropy in the MCS and the FCS entails different dispersion relations according to the principal direction **e**_*n*_ with *n* ∈ {1, 2, 3}. However, in each principal direction, a band gap is opened for the MCS in the frequency range where the effective bulk modulus has negative values. This band gap is characterised by quasi non-propagative waves, ${\rm{Re}}({k}_{n}^{MCS})\to 0$, with characteristic attenuation length 1/${\rm{Im}}({k}_{n}^{MCS})$ much shorter than the wavelength 2*π*/${\rm{Re}}({k}_{n}^{MCS})$, and vanishing sound speed ${\rm{Re}}({c}_{n}^{MCS})\to 0$.

At frequencies below the band gap, the wave speeds are much smaller than the wave speed in both the FSC and the air, resulting in a slow sound regime. In this regime, the wave speeds possess a plateau at low frequencies and slowly decrease to a value close to zero at the frequency of the lower bound of the band gap. It is worth noting that the group velocity, defined as ${v}_{g}=\partial \omega$/$\partial {k}_{n}^{MCS}$ is also much lower than the group velocity in both the FCS and in air and tend to zero at the frequency of the lower bound of the band gap. We notice also that the inherent losses of the MCS, translating in Im(*k*_*n*_), are not negligible for the frequencies below the band gap. At frequencies above the band gap the wave speed is higher than that of both the FSC and the air, resulting in a supersonic regime^19,21,22^.

Conversely, the wave dispersion and attenuation in the FCS are weak in every direction: the sound speed along the principal directions are almost constant in the inertial-adiabatic regime above 100 Hz, with the sound speed being close to that of air in the direction **e**_1_ but almost divided by 2 along the directions **e**_2_ due to a tortuosity effect. For both the FCS and the MCS, the sound speed decreases down to zero as *ω* → 0 in the viscous regime below 100 Hz.

## Critical Coupling and Perfect Absorption

The slow sound effect and the inherent losses of the MCS discussed before can be used to design finite size perfect absorber samples. On the one hand, the frequencies of the Fabry-Perot resonances drastically decrease due to the slow sound regime of the MCS. As a consequence, these resonances happen for wavelengths much larger than the structure thickness, i.e., the MCS becomes deeply sub-wavelength. On the other hand, the MCS layer behaves as an open lossy and resonant system, characterised at the resonant frequencies, by both the leakage rate of energy (i.e., the coupling of the resonant elements with the propagating medium) and the inherent losses. In the reflection problem, the balance between the leakage and the losses activates the condition of critical coupling, enabling a perfect impedance matching to the background medium, and therefore generating a perfect energy absorption^12,22,23^. The layer sample analysed here and depicted in Fig. 3 has been designed so that the layer of thickness *L* = 13${\ell }_{3}$ = 109.2 mm is critically coupled to air under plane wave excitation at normal incidence with *λ*_*e*_ = 13 *L* in the principal direction **e**_3_, see Section Methods.

In a reflection problem, the representation of the zeros and the poles of the reflection coefficient in the complex frequency plane is an efficient tool to interpret the critical coupling condition^12^. Introducing the complex frequency plane (complex frequency $\tilde{\omega }=\omega +{\rm{i}}{\omega }_{{\rm{Im}}}$), the reflection coefficient, $R(\tilde{\omega })$, represents the scattering of the system. In the lossless case, the relation ${R}^{\ast }(\tilde{\omega })=1$/$R({\tilde{\omega }}^{\ast })$ is satisfied due to temporal invariance symmetry, where ${R}^{\ast }(\tilde{\omega })$ and ${\tilde{\omega }}^{\ast }$ are the complex conjugate of $R(\tilde{\omega })$ and $\tilde{\omega }$ respectively. Consequently, the reflection coefficient possesses pairs of poles and zeros at complex frequencies being complex conjugate with each other. More importantly, the imaginary part of the complex frequency of the pole represents the energy leakage of the system. In the lossy case, when inherent losses are introduced, the zeros and poles are not complex conjugates any more and in particular both are shifted downward in the complex frequency plane, if *e*^−i*ωt*^ is used. The zero of the reflection coefficient can thus intersect the real frequency axis, enabling perfect absorption when exactly located on this axis.

**Figure 6:**
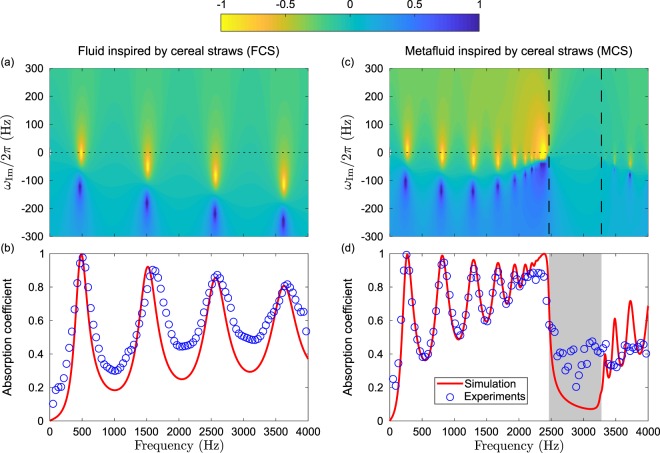
Complex frequency planes representing log(|*R*|^2^) for the FCS (**a**) and for the MCS (**c**). Absorption coefficients (theoretical prediction in continuous red line, experiments in open circles) for the FCS (**b**) and for the MCS (**d**).

Figure 6(a) shows the complex frequency plane for the FCS sample. The pairs of pole/zero represent the different Fabry-Perot resonances of the system. Complementary, Fig. 6(b) depicts the theoretical predictions and the experimental results of the absorption coefficient for the FCS sample. Both the pairs of pole/zero and the absorption peaks appear almost equally spaced in frequency, approximatively following the typical harmonic series {1, 3, 5, 7, …} of the quarterwavelength resonances of a rigidly backed layer, because the system is weakly dispersive (as shown in Fig. 5(a,b)). Moreover, the distance of the zero of the reflection coefficient to the real frequency axis is related to the amplitude of the absorption peaks: the closer the zero to the real frequency axis, the higher the absorption peak. Notably, a near-perfect absorption (97.4%) is observed experimentally on the first peak at 509 Hz. Experimental results are in very good agreement with the theoretical predictions.

Figure 6(c,d) respectively show the complex frequency plane and the absorption coefficient for the MCS sample. The absorption peaks and the pairs pole/zero are not any more equally spaced in frequency due to the strong dispersion introduced by the resonances of the double QWR. Here, we pay attention to the frequencies below the band gap. The first absorption peak appears at 272 Hz. The ratio of the wave speed between the FCS and the MCS along the **e**_2_ (=**e**_3_) direction [in Fig. 5(c)] is in good agreement with the downshifting of this peak at low frequencies with respect to the first absorption peak of the FCS. More importantly, we observe perfect absorption for this peak (experimentally 99.6% at 258 Hz), where the corresponding wavelength equals to 13 times the slab thickness. Thus, this system represents a sub-wavelength perfect absorber. A cascade of nearly perfect absorption peaks, due to the higher order Fabry-Perot resonances, is produced and perfectly grasper by the theory. Each of these nearly perfect absorption peak is associated with a low quality factor resonance arising from the coupling with viscous and thermal diffusion tailored by the concentration of double QWR and the corrugation. These low quality factors strongly contribute to enhance the absorption between two consecutive peaks. Nevertheless, a perfect absorption peak theoretically predicted at the lower bound of the band gap around 2.39 kHz is in disagreement with the experimental data, in which an absorption coefficient of 0.86 is achieved. This is due to the fact that the theoretical model assumes an infinite number of the resonators along the structure depth, creating an accumulation point^24^, while this number is finite in practice. Effectively, the MCS sample comprises only 13 unit cells along the **e**_3_ direction.

## Conclusions

The metafluid inspired by cereal straws has been theoretically and experimentally reported for sub-wavelength perfect sound absorption. A straw bale constituted of straws cut on either side of a node is idealized by a three-dimensional periodic repetition of tightly packed double QWR. This idealised straw bale is homogenised, exhibiting slow sound region enabling to increase of the density of state in the subwavelength regime together with viscous and thermal diffusion through the arrangement of stems. The MCS behaves like an open and lossy resonator, the absorption properties of which are tailored by critical coupling. Therefore, the designed MSC is a sub-wavelength perfect absorber. Moreover, the strong dispersion arising from the fundamental resonance of the double QWR induces a cascade of nearly perfect absorption peaks below the lower bound of the band gap. The absorption between consecutive peaks is strongly enhanced by the viscous and thermal diffusion through the anisotropic surrounding fluid. The designed MCS possess a 99.6% absorption peak at 258 Hz, which corresponds to a wavelength 13 times larger than of the slab thickness. The design of the present MSC offers both a comprehensive explanation of the acoustic properties of bio-sourced materials, such as wheat, reed, or rattan and large perspectives for the design of bio-inspired metafluid, notably through its anisotropy.

## Methods

The density ${\rho }_{e}=1.213$ kg/m^3^, adiabatic constant $\gamma =1.4$, thermal conductivity $\kappa =2.5\times {10}^{-2}$ W/m/K, isobaric heat capacity ${C}_{p}=1.219\times {10}^{3}$ J/K, and viscosity $\eta =1.839\times {10}^{-5}$ Pa.s were used for air at equilibrium, as well as the atmospheric pressure ${P}_{e}=1.013\times {10}^{5}$ Pa, the bulk modulus ${B}_{e}=\gamma {P}_{e}$, the sound speed ${c}_{e}=\sqrt{{B}_{e}/{\rho }_{e}}$ and the air wavenumber ${k}_{e}=\omega$/*c*_*e*_.

### Poro-acoustics with inner resonances

Using the theory of poro-acoustics with inner resonances^19^, the effective pressure *P* and particle velocity **V** in the MCS are governed by the following equations of mass conservation and generalised Darcy law,

6 $$\nabla \cdot {\bf{V}}={\rm{i}}\omega \frac{{\varphi }_{0}{\beta }_{0}}{{B}_{e}}P+\frac{{Q}_{1}+{Q}_{2}}{|{\rm{\Omega }}|},\,{\bf{V}}=-\,\frac{{\bf{K}}}{\eta }\nabla P,$$

where *Q*_*j*_ is the flux pulsed out from the aperture ${{\rm{\Sigma }}}_{j}$, and where the compressibility factor *β*_0_ in ${{\rm{\Omega }}}_{0}$ is driven by the thermal permeability ${\rm{\Theta }}$ according to,

7 $${\beta }_{0}=\gamma +{\rm{i}}\omega \,(\gamma -1)\,{\rm{\Theta }}\,{C}_{p}/({\varphi }_{0}\kappa ).$$

Equation (6) reveals that the QWRs act as secondary acoustic sources in the mass conservation: they radiate the flux ${Q}_{1}+{Q}_{2}$ in the effective medium formed by the array of straws, as these latter modify the air flow path. Conversely to the case of FCS for which ${Q}_{1}={Q}_{2}=0$, the presence of the nodes within the straws makes each duct ${{\rm{\Omega }}}_{j}$ behave as a QWR emitting the flux,

8 $${Q}_{j}=\frac{|{{\rm{\Sigma }}}_{j}|P}{1/{Y}_{j}+{\rm{i}}\omega {\rho }_{e}\mu {r}_{i}}\,{\rm{with}}\,{Y}_{j}=\frac{{\rm{i}}\omega \,\tan ({k}_{j}{h}_{j})}{{k}_{j}{B}_{j}},$$

where $|{{\rm{\Sigma }}}_{j}|=|{{\rm{\Omega }}}_{j}|$/*h*_*j*_ is the surface area of the apertures, *Y*_*j*_ is the surface admittance at the aperture ${{\rm{\Sigma }}}_{j}$. Substitution of Eq. (8) into (6) results in the equations of effective mass conservation and Darcy law in the MCS presented in Eq. (1). Furthermore, the effective density and bulk modulus of air in the ducts ${{\rm{\Omega }}}_{j}$ with $j\in \{1,2\}$ satisfy Zwikker & Kosten model^17^:

9 $$\frac{{\rho }_{j}}{{\rho }_{e}}=-\,\frac{{{\bf{J}}}_{0}({X}_{v})}{{{\bf{J}}}_{2}({X}_{v})},\,\frac{{B}_{e}}{{B}_{j}}=\gamma +(\gamma -1)\frac{{{\bf{J}}}_{2}({X}_{t})}{{{\bf{J}}}_{0}({X}_{t})},$$

where **J**_0_ and **J**_2_ are Bessel functions of the first kind and order 0 and 2, while ${X}_{v}={r}_{i}\sqrt{{\rm{i}}\omega {\rho }_{e}/\eta }$ and ${X}_{t}={r}_{i}\sqrt{{\rm{i}}\omega {C}_{p}/\kappa }$ represent the duct radius normalized by the viscous and thermal skin-depths. Thermal permeability ${\rm{\Theta }}$ and principal Darcy permeabilities *K*_*n*_ with $n\in \{1,2,3\}$, are given by Johnson formula^13,16^,

10 $${K}_{n}={K}_{n}^{0}/(\sqrt{1-{\rm{i}}\omega {(2{\delta }_{n}/{{\rm{\Lambda }}}_{n})}^{2}/{\omega }_{n}}-{\rm{i}}\omega /{\omega }_{n}),$$

11 $${\rm{\Theta }}={{\rm{\Theta }}}^{0}/(\sqrt{1-{\rm{i}}\omega {(2{\delta }_{\theta }/{{\rm{\Lambda }}}_{\theta })}^{2}/{\omega }_{\theta }}-{\rm{i}}\omega /{\omega }_{\theta }),$$

where ${\omega }_{n}$ and ${\omega }_{\theta }={\mathscr{O}}({\omega }_{n})$ are characteristic viscous and thermal frequencies, ${\delta }_{n}=\sqrt{\eta /({\rho }_{e}{\omega }_{n})}$ and ${\delta }_{\theta }=\sqrt{\kappa /({C}_{p}{\omega }_{\theta })}$ are viscous and thermal skin depths at these characteristic frequencies, ${{\rm{\Lambda }}}_{n}$ and ${{\rm{\Lambda }}}_{\theta }$ are characteristic viscous and thermal lengths and ${K}_{n}^{0}$ and ${{\rm{\Theta }}}^{0}$ are the static values of *K*_*n*_ and ${\rm{\Theta }}$ as $\omega \to 0$. The characteristic frequencies ${\omega }_{n}$ and ${\omega }_{\theta }$ are defined by:

12 $${\omega }_{n}={\varphi }_{0}\eta /({K}_{n}^{0}{\rho }_{e}{\tau }_{n}^{\infty }),\,{\omega }_{\theta }={\varphi }_{0}\kappa /({{\rm{\Theta }}}^{0}{C}_{p}),$$

where ${\tau }_{n}^{\infty }$ is the high frequency tortuosity related to *K*_*n*_.

### Cell problems

Following two-scale asymptotic homogenisation^14,15^, the parameters ${{\rm{\Theta }}}^{0}$ and ${{\rm{\Lambda }}}_{\theta }$ characterising the thermal behaviour, and ${K}_{n}^{0}$, ${{\rm{\Lambda }}}_{n}$ and ${\tau }_{n}^{\infty }$ characterising the visco-inertial behaviour are given as volume and surface averages of ${\rm{\Omega }}$-periodic tests fields *θ*, **u**_*n*_ and *χ*_*n*_ defined on the air domain ${{\rm{\Omega }}}_{0}$:

13a $${{\rm{\Theta }}}^{0}=\frac{1}{|{\rm{\Omega }}|}\mathop{\int }\limits_{{{\rm{\Omega }}}_{0}}\,\theta \,{\rm{d}}{\rm{\Omega }},\,{K}_{n}^{0}=\frac{1}{|{\rm{\Omega }}|}\,\mathop{\int }\limits_{{{\rm{\Omega }}}_{0}}\,{{\bf{u}}}_{n}\cdot {{\bf{e}}}_{n}\,{\rm{d}}{\rm{\Omega }},$$

13b $${{\rm{\Lambda }}}_{\theta }=2\frac{|{{\rm{\Omega }}}_{0}|}{|{{\rm{\Gamma }}}_{0}|},\,{{\rm{\Lambda }}}_{n}=2\frac{{\int }_{{{\rm{\Omega }}}_{0}}{({{\bf{e}}}_{n}-\nabla {\chi }_{n})}^{2}\,{\rm{d}}{\rm{\Omega }}}{{\int }_{{{\rm{\Gamma }}}_{0}}{({{\bf{e}}}_{n}-\nabla {\chi }_{n})}^{2}\,{\rm{d}}{\rm{\Gamma }}},$$

13c $${\tau }_{n}^{\infty }=|{{\rm{\Omega }}}_{0}|/\,\mathop{\int }\limits_{{{\rm{\Omega }}}_{0}}1-\nabla {\chi }_{n}\cdot {{\bf{e}}}_{n}\,{\rm{d}}{\rm{\Omega }},$$

where *θ*, **u**_*n*_ and *χ*_*n*_ satisfy the following cell problems, with *χ*_*n*_ and ${\zeta }_{n}$ having zero mean values over ${{\rm{\Omega }}}_{0}$:

14a $${\rm{\Delta }}\theta =-\,1,\,\theta =0\,{\rm{on}}\,{{\rm{\Gamma }}}_{0},$$

14b $${\rm{\Delta }}{{\bf{u}}}_{n}=\nabla {\zeta }_{n}-{{\bf{e}}}_{n},\,\nabla \cdot {{\bf{u}}}_{n}=0,\,{{\bf{u}}}_{n}=0\,{\rm{on}}\,{{\rm{\Gamma }}}_{0},$$

14c $${\rm{\Delta }}{\chi }_{n}=0,\,(\nabla {\chi }_{n}-{{\bf{e}}}_{n})\cdot {\bf{n}}=0\,{\rm{on}}\,{{\rm{\Gamma }}}_{0}.$$

In Eqs (13) and (14), vector **n** is normal to the boundary ${{\rm{\Gamma }}}_{0}={{\rm{\Sigma }}}_{1}\cup {{\rm{\Sigma }}}_{2}\cup {{\rm{\Sigma }}}_{{\rm{lat}}}$ where ${{\rm{\Sigma }}}_{{\rm{lat}}}$ is the lateral surface of the straws. The cell problems (14) are computed numerically by means of the Finite Element Method using commercial software COMSOL Multiphysics®. The computations are performed in 3-D on a single unit cell with periodicity conditions. Problems in Eq. (14a) and (14c) are solved with module *Coefficient Form PDE* in *Mathematics* while problem in Eq. (14b) is solved with module *Creeping flow* in *Fluid Flow*. The geometry has been meshed with Free Tetrahedral elements, sufficiently small to ensure accuracy. To account for the corrugation on the cell problems, similar cell problems are computed for the square array of infinite cylinders with corrugated lateral surface and values of the tests-fields hence obtained are substituted into Eq. (13) in the domain between the cylinders, while values of *θ*, **u**_*n*_ and *χ*_*n*_ in the air gap of thickness ${\ell }_{1}-h$ in the direction **e**_1_ is kept unchanged. A corrugation profile in the form of semi-ellipses in a radial cross-section is considered in the computations. It leads to:

15 $$\{\begin{array}{lll}{\varphi }_{0}=0.284, & {{\rm{\Theta }}}^{0}=0.11\,{{\rm{mm}}}^{2}, & {{\rm{\Lambda }}}_{\theta }=1.1\,{\rm{mm}},\\ {\tau }_{1}^{\infty }=1.06, & {K}_{1}^{0}=0.11\,{{\rm{mm}}}^{2}, & {{\rm{\Lambda }}}_{1}=1.4\,{\rm{mm}},\\ {\tau }_{2}^{\infty }=2.07, & {K}_{2}^{0}=0.004\,{{\rm{mm}}}^{2}, & {{\rm{\Lambda }}}_{2}=0.4\,{\rm{mm}},\end{array}$$

for both MCS and FCS analysed in this work. To emphasize the effects of the corrugation, the parameters calculated for the smooth surface (${h}_{c}=0$) were:

16 $$\{\begin{array}{lll}{\varphi }_{0}=0.30, & {{\rm{\Theta }}}^{0}=0.14\,{{\rm{mm}}}^{2}, & {{\rm{\Lambda }}}_{\theta }=1.6\,{\rm{mm}},\\ {\tau }_{1}^{\infty }=1.06, & {K}_{1}^{0}=0.14\,{{\rm{mm}}}^{2}, & {{\rm{\Lambda }}}_{1}=1.6\,{\rm{mm}},\\ {\tau }_{2}^{\infty }=2.07, & {K}_{2}^{0}=0.007\,{{\rm{mm}}}^{2}, & {{\rm{\Lambda }}}_{2}=0.7\,{\rm{mm}}.\end{array}$$

### Samples and experimental set-up

The reflection of a plane wave from a layer of material of thickness *L* arranged against a rigid backing is studied. The air/layer interface ${{\rm{\Gamma }}}_{L}^{0}$ is located at ${x}_{3}=0$, and the rigid backing ${{\rm{\Gamma }}}_{L}^{b}$ at ${x}_{3}=-\,L$. Both interfaces have the normal vector **e**_3_ which is principal direction of the Darcy tensor. Moreover, normal incidence is considered, so that the pressure reflection coefficient and absorption coefficient read as $R=(1-\Upsilon )$/$(1+\Upsilon )$ and ${\mathscr{A}}=1-|R{|}^{2}$ where the normalised admittance $\Upsilon$ of the layer reads as follows,

17 $$\Upsilon =-\,{\rm{i}}\,[{B}_{e}{c}_{3}/(B{c}_{e})]\,\tan (\omega L/{c}_{3}).$$

The MCS geometrical parameters were tuned using optimisation Sequential Quadratic Programming. The minimised cost function is |*R*|^2^ at the required frequency. The MCS and FCS samples were fabricated using a MakerBot ®Replicator ®2X Experimental 3D Printer based on the Fused Filament Fabrication technology. Heated continuous filament of thermoplastic material is deposited layer-by-layer by the extruder head having a 400 *μ*m diameter nozzle. The layer height setting is 200 *μ*m, which corresponds to the period measured on the ESM cliché in Fig. 3. Impedance tube measurements were performed in a tube with 4.2 cm × 4.2 cm square cross section. Assuming that plane waves propagate below the cut-off frequency of the tube (4200 Hz), the walls of the tube act as perfect mirrors to emulate a periodicity pattern in the directions **e**_1_ and **e**_2_. The sample is placed at the end of the tube against a copper plug that closes the tube. A single microphone (1/4 inch pressure field B&K microphone type 4938) attached on a one-dimensional robotised arm is used. This technique provides the experimental absorption coefficient at normal incidence without cross-calibration of many microphones and with an enhanced accuracy at low frequencies^21,22^. Input sine signals were generated and output signals from microphone were acquired by the two-channel dynamic signal analyzer (Stanford Research Systems model SR785).

## References

[1] MacDonald E, Wicker R (2016). Multiprocess 3d printing for increasing component functionality. Science.

[2] Kadic M, Bückmann T, Stenger N, Thiel M, Wegener M (2012). On the practicability of pentamode mechanical metamaterials. Appl. Phys. Lett..

[3] Jiménez N, Romero-García V, Pagneux V, Groby J-P (2017). Rainbow-trapping absorbers: Broadband, perfect and asymmetric sound absorption by subwavelength panels for transmission problems. Scientific Reports.

[4] Shen C, Díaz-Rubio A, Li J, Cummer SA (2018). A surface impedance-based three-channel acoustic metasurface retroreflector. Appl. Phys. Lett..

[5] Affolderbach C (2018). Study of additive manufactured microwave cavities for pulsed optically pumped atomic clock applications. Appl. Phys. Lett..

[6] Vukusic P, Sambles JR (2003). Photonic structures in biology. Nature.

[7] Miniaci M, Krushynska A, Movchan AB, Bosia F, Pugno NM (2016). Spider web-inspired acoustic metamaterials. Appl. Phys. Lett..

[8] Khalil ISM, Dijkslag HC, Abelmann L, Misra S (2014). Magnetosperm: A microrobot that navigates using weak magnetic fields. Appl. Phys. Lett..

[9] Oldham DJ, Egan CA, Cookson RD (2011). Sustainable acoustic absorbers from the biomass. Appl. Acoust..

[10] Verdière K, Panneton R, Elkoun S (2016). Prediction of the acoustic behavior of a parallel assembly of hollow cylinders. Appl. Acoust..

[11] Luu HT, Perrot C, Monchiet V, Panneton R (2017). Three-dimensional reconstruction of a random fibrous medium: Geometry, transport, and sound absorbing properties. J. Acoust. Soc. Am..

[12] Romero-Garca V, Theocharis G, Richoux O, Pagneux V (2016). Use of complex frequency plane to design broadband and sub-wavelength absorbers. J. Acoust. Soc. Am..

[13] Johnson DL, Koplik J, Dashen R (1987). Theory of dynamic permeability and tortuosity in fluid-saturated porous media. Journal of fluid mechanics.

[14] Sanchez-Palencia, E. *Non-Homogeneous Media and Vibration Theory*, vol. 127 of *Lecture Notes in Physics*. (Springer-Verlag, Berlin, Heidelberg, 1980).

[15] Auriault, J.-L., Boutin, C. & Geindreau, C. *Homogenization of Coupled Phenomena in Heterogenous Media*. (ISTE Ltd and Wiley, 2009).

[16] Lafarge D, Lemarinier P, Allard JF (1997). Dynamic compressibility of air in porous structures at audible frequencies. J. Acoust. Soc. Am..

[17] Zwikker, C. & Kosten, C. W. *Sound absorbing materials*. (Elsevier Publishing Company, 1949).

[18] Fang N (2006). Ultrasonic metamaterials with negative modulus. Nature materials.

[19] Boutin C (2013). Acoustics of porous media with inner resonators. J. Acoust. Soc. Am..

[20] Torrent, D. & Sánchez-Dehesa, J. Sound scattering by anisotropic metafluids based on two-dimensional sonic crystals. *Phys*. *Rev*. *B* **17** (2009).

[21] Groby J-P, Huang W, Lardeau A, Aurégan Y (2015). The use of slow waves to design simple sound absorbing materials. J. Appl. Phys..

[22] Groby, J.-P., Pommier, R. & Aurégan, Y. Use of slow sound to design perfect and broadband passive sound absorbing materials,. *J*. *Acoust*. *Soc*. *Am*. **139** (2016). 10.1121/1.4945101 27106313

[23] Jiménez N, Huang W, Romero-García V, Pagneux V, Groby J-P (2016). Ultra-thin metamaterial for perfect and quasi-omnidirectional sound absorption. Appl. Phys. Lett..

[24] Jiménez N, Romero-Garca V, Pagneux V, Groby J-P (2017). Quasiperfect absorption by subwavelength acoustic panels in transmission using accumulation of resonances due to slow sound. Phys. Rev. B.

